# Prevention and management of metabolic bone disease of prematurity: a systematic review

**DOI:** 10.1038/s41372-026-02781-z

**Published:** 2026-06-29

**Authors:** L. M. Alghawas, M. Buck, C. Conway, C. Cunningham, K. El-Hanan, R. Hession, N. Karadede, J. A. Malone, O. E. Oce, E. O’Kane, D. Petsis, M. Volchok Wynia, J. Trayer, P. Stewart, R. McCarthy, E. F. Roche, Sara Kift, J. Meehan, E. J. Molloy

**Affiliations:** 1 Discipline of Paediatrics, Trinity College Dublin, The University of Dublin, Dublin, Ireland; 2 Trinity Translational Medicine Institute (TTMI), St James Hospital, Dublin, Ireland; 3 Trinity Research in Childhood Centre (TRiCC), Dublin, Ireland; 4 Department of Paediatric Endocrinology and Diabetes, Children’s Health Ireland, Tallaght, Dublin, Ireland; 5 Paediatrics, Coombe Hospital, Dublin, Ireland; 6 Neurodisability, Children’s Health Ireland (CHI), Dublin, Ireland; 7 Neonatology, Children’s Health Ireland (CHI), Dublin, Ireland

**Keywords:** Metabolic disorders, Outcomes research

## Abstract

**Objective:**

Metabolic Bone Disease of Prematurity (MBDP) results from disrupted third-trimester mineral accretion in preterm neonates, leading to inadequate bone mineralization. Despite adherence to standard nutritional guidelines, MBDP remains common. This systematic review assesses the effectiveness of nutritional supplementation and physiotherapy in reducing MBDP incidence and severity.

**Study design:**

The review was conducted under PRISMA guidelines and registered with PROSPERO. We retrieved 942 studies from PubMed and Embase. Eligible studies included RCTs/cohort studies published 2020–2025 that investigated postnatal calcium, phosphate, vitamin D supplementation, or physiotherapy in preterm infants (<37 weeks), while recognising that the highest MBDP burden occurs in infants born <32 weeks or with very low birth weight and that selected moderate-to-late preterm infants may remain vulnerable when additional nutritional or clinical risk factors are present. Studies were screened with Covidence, and relevant data were qualitatively synthesized by intervention type and outcomes.

**Results:**

From a total of 942 records initially identified, sixteen studies were included (eleven supplementation, five physiotherapy). Higher-dose calcium and organic phosphate, especially when administered continuously, improved biochemical and radiological outcomes. High-dose vitamin D offered no benefit over standard dosing, but individualized monitoring improved safety. Limited evidence demonstrated that active or reflex-based physiotherapy improved bone density and growth but with some concerns for safety; passive approaches had limited effect.

**Conclusion:**

Continuous high-dose calcium and organic phosphate are particularly beneficial. Standardized diagnostic criteria for biochemical and radiological MBDP assessment are needed, as the absence of consensus disease definitions remains a major barrier to interstudy comparability, evidence synthesis, and the development of clinical intervention protocols.

**Registration:**

This systematic review was prospectively registered with PROSPERO (CRD420251116486).

## Introduction

Metabolic Bone Disease of Prematurity (MBDP) is a multifactorial condition characterised by impaired bone mineralisation in preterm infants born before 37 weeks’ gestation, particularly those born before 32 weeks’ gestation or with a very low birth weight (VLBW, <1500 g) or an extremely low birth weight (ELBW, <1000 g) [1]. Incidence rates remain high, affecting up to 32% of VLBW and 54% of ELBW infants [2], with greater frequency in those born before 28 weeks [3]. The <37-week threshold was therefore used in this review as an inclusive population definition rather than a statement of equal disease risk across all gestations. While MBDP predominantly affects very preterm infants, those born between 32 and 37 weeks may still warrant biochemical monitoring when significant nutritional compromise or prolonged neonatal morbidity is present.

Interchangeable terms are used in the literature, including “osteopenia of prematurity”, “neonatal bone disease”, and “neonatal rickets”; this review will refer to the term MBDP in reference to current guidance of the American Academy of Paediatrics (AAP) and the European Society for Paediatric Gastroenterology, Hepatology and Nutrition (ESPGHAN) [4, 5].

MBDP arises from the disruption of mineral accretion that normally occurs during the third trimester, when 80% of foetal calcium and phosphorus transfer occurs via active placental transport [5, 6]. In utero, mineral accretion rates peak between 28–36 weeks at approximately 120 mg/kg/day (3.0 mmol/kg/day) for calcium and 60 mg/kg/day (1.9 mmol/kg/day) for phosphorus, supported by high oestrogen and calcitonin with low PTH, which together promote foetal bone mineral deposition and suppress excessive bone resorption [4]. Early delivery interrupts this process, resulting in inadequate foetal mineral reserves at birth and abrupt loss of placental calcium supply, which may trigger secondary hyperparathyroid activity, urinary phosphate wasting, and skeletal mineral mobilisation. Reduced mechanical loading after preterm birth may impair osteoblastic bone formation and favour bone resorption, resulting in net bone loss [7, 8].

Postnatal risk factors further exacerbate this mineral deficit, including delayed or insufficient enteral feeding, prolonged parenteral nutrition (PN) with inherent mineral solubility limitations, vitamin D deficiency in select high-risk populations, immobility due to clinical instability or mechanical ventilation, chronic lung disease, sepsis, cholestasis, and medications such as loop diuretics and glucocorticoids particularly when administered over prolonged neonatal intensive care courses, as these agents increase renal calcium wasting, suppress osteoblastic proliferation, reduce intestinal calcium absorption, and promote bone resorption; methylxanthines such as caffeine have also been implicated as contributory risk factors [1–3, 5, 8, 9]. PN often fails to match intrauterine mineral accretion rates due to risks of calcium–phosphorus precipitation and physicochemical incompatibilities [5, 9]. Sepsis and bronchopulmonary dysplasia prolong PN use, as septic infants commonly experience haemodynamic instability and feed intolerance while infants with bronchopulmonary dysplasia frequently require prolonged respiratory support, fluid restriction, and delayed advancement of enteral nutrition, all of which limit adequate mineral delivery and extend dependence on parenteral supplementation, increasing MBDP risk [10]. Cholestasis impairs vitamin D absorption and inhibits osteoblast proliferation [10].

AAP and ESPGHAN recommend early nutritional strategies, including fortified human milk or preterm formula, with targeted supplementation of calcium, phosphorus, and vitamin D; however, recommended intake ranges differ slightly between societies [4, 5, 9]. Both advocate maintenance of balanced calcium-to-phosphorus delivery and routine vitamin D supplementation in high-risk preterm infants. The recommended intake ranges are summarised in

Evidence for adjunctive strategies, such as higher-dose mineral supplementation beyond guideline recommendations or physiotherapy interventions to enhance mechanical bone loading, remains limited and has not been synthesised jointly in systematic reviews. Existing reviews have predominantly evaluated nutritional and physiotherapy interventions without direct comparison.

This systematic review aims to evaluate the effectiveness of enhanced nutritional supplementation, including calcium, phosphate, and vitamin D, beyond standard practice recommendations and/or physiotherapy-based interventions, compared with current standard care or no intervention for the prevention and management of MBDP.

## Methods

### Review framework

This systematic review was conducted in accordance with the Preferred Reporting Items for Systematic reviews and Meta-Analyses (PRISMA) 2020 guidelines [11]. The objective was to evaluate the management strategies for MBDP, focusing on postnatal interventions for prevention and treatment.

### Review design and process

A systematic review methodology was used, with Covidence software used for study screening, eligibility assessment, and data extraction. The review process comprised two screening stages: initial title and abstract screening, followed by full-text review.

**Fig. 1:**
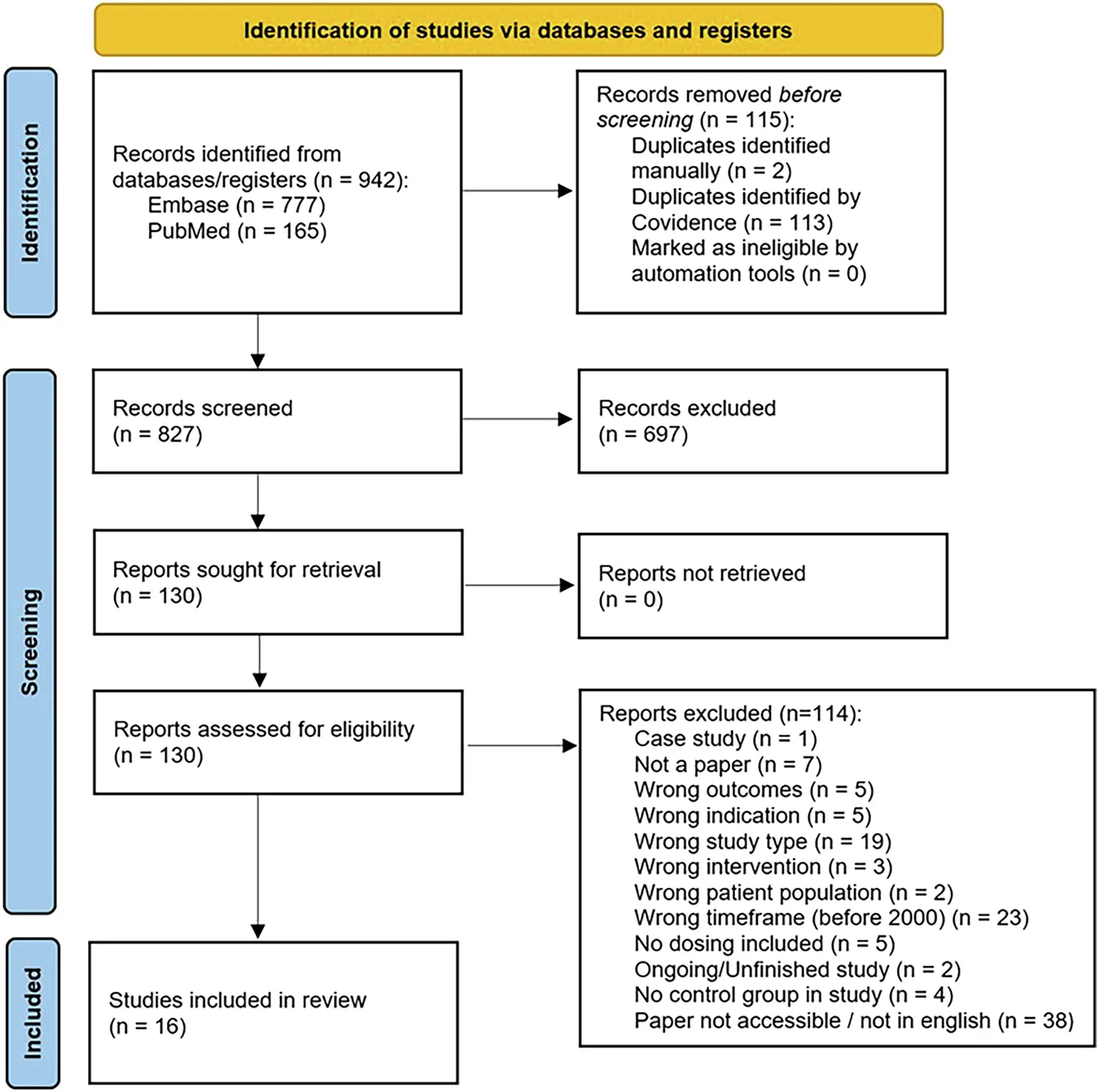
PRISMA 2020 flow diagram summarising the study selection process, including numbers identified, screened, assessed for eligibility, and included. The 697 records excluded at screening did not meet predefined inclusion criteria.

A total of 942 records were identified (777 from Embase, 165 from PubMed). After removing 115 duplicates (113 automatic, 2 manual), 827 records remained for screening, conducted independently by 12 reviewers. Of these, 130 full texts were assessed for eligibility. Although studies before 2000 were excluded by eligibility criteria, some were retrieved automatically during database searches and excluded at full-text review. Sixteen studies met inclusion criteria. Data extraction was performed independently by six reviewers, with discrepancies resolved by discussion and consensus. The study selection process is summarised in the PRISMA 2020 flow diagram (Fig. 1).

### Eligibility Criteria

The inclusion criteria were developed using the PICO framework (Population, Intervention, Comparison, Outcome). The population consisted of preterm infants born before 37 weeks’ gestation, at increased risk of or diagnosed with MBDP, also referred to as osteopenia of prematurity (OFP), neonatal bone disease, or neonatal rickets. Interventions included postnatal management strategies such as nutritional supplementation with calcium, phosphate, and vitamin D, and physiotherapy including structured physical therapy, massage, reflex therapy, and passive range-of-motion exercises. The comparison group included standard care, defined as routine neonatal nutritional management and biochemical surveillance according to local NICU feeding and supplementation protocols, no intervention, defined as absence of any additional study-specific supplementation or physiotherapy beyond baseline neonatal care, or delayed intervention, defined as later initiation of supplementation, fortification, or physiotherapy after the comparator group or only after biochemical or radiological evidence of MBDP had emerged. Primary outcomes were biochemical markers principally serum alkaline phosphatase [ALP], serum phosphate, and serum calcium, with additional biochemical markers including parathyroid hormone, 25-hydroxyvitamin D, urinary mineral indices, tubular phosphate reabsorption, and bone turnover markers extracted where available, and radiological measures assessed by DEXA scan or X-ray. Ultrasonography was considered during data extraction but was not used as a comparative bone assessment modality in the included intervention studies. DEXA outcomes were accepted where studies reported bone mineral content, bone mineral density, or bone area according to individual study protocols, as no uniform neonatal DEXA reference standard was consistently applied across the literature. Secondary outcomes were fracture incidence and growth parameters, although fracture reporting was inconsistent across included studies and did not permit reliable subdivision into long-bone versus rib fractures for comparative synthesis.

### Study characteristics

Eligible studies were peer-reviewed, published in English from 2000, and designed as RCTs or observational cohorts. Studies had to describe postnatal nutritional or physiotherapy interventions for MBDP, include dosing details, and have control groups where appropriate.

The exclusion criteria were: publication in a language other than English; publication before 2000; ineligible study design (case reports, case series, systematic reviews, editorials, commentaries); ongoing or incomplete studies; adult participants or unclear gestational age criteria; antenatal interventions; lack of relevant bone-related outcomes; absence of treatment-specific or dosing information Table 1; lack of full-text availability; non–peer-reviewed studies; or clinical indications unrelated to treatment or prevention of MBDP.

**Table 1:** Despite adherence to these guidelines, MBDP remains prevalent, indicating that standard nutritional supplementation alone may be insufficient.

Society	Calcium	Phosphorus	Vitamin D
AAP	150–220 mg/kg/day (3.7–5.5 mmol/kg/day)	75–140 mg/kg/day (2.4–4.5 mmol/kg/day)	200–400 IU/day; increase to 400 IU/day once >1500 g and stable on enteral feeds
ESPGHAN	120–200 mg/kg/day (3.0–5.0 mmol/kg/day)	70–115 mg/kg/day (2.3–3.7 mmol/kg/day)	400–700 IU/day (maximum 1000 IU/day)

Recommended calcium, phosphorus, and vitamin D intake ranges for preterm infants according to AAP and ESPGHAN guidelines.

Both societies recommend early nutritional support with fortified human milk or preterm formula. ESPGHAN additionally recommends a calcium-to-phosphorus ratio of 1.5–1.7:1.

### Search strategy and information sources

PubMed and Embase were searched using MeSH and free-text terms: (“metabolic bone disease” OR “osteopenia of prematurity” OR “neonatal bone disease”) AND (“preterm infants” OR “premature neonates”) AND (“management” OR “treatment” OR “supplementation” OR “therapy” OR “nutritional supplementation” OR “calcium and phosphate supplementation” OR “vitamin D”).

### Data extraction and synthesis

Data were extracted using a standard template, including study design, population characteristics, interventions, control group type, inclusion and exclusion criteria, outcomes, results, and effect sizes or confidence intervals. Studies were categorised as nutritional supplementation or physiotherapy interventions. Due to heterogeneity in study design, meta-analysis was not conducted. Results were qualitatively combined and grouped by intervention type and outcomes. Most included studies were RCTs or prospective cohort studies. Detailed study characteristics are provided in Supplementary Table 1.

## Results

Sixteen studies met inclusion criteria, encompassing 1191 preterm infants. Eleven studies investigated nutritional or supplementation interventions, while five evaluated physiotherapy-based strategies for the prevention or management of MBDP. Primary outcomes included biochemical markers of bone metabolism, most commonly serial alkaline phosphatase, serum phosphate, serum calcium, parathyroid hormone, urinary mineral handling indices, and selected bone turnover markers, which were used across studies as early surrogate indicators of impaired mineralisation or treatment response. However, the biochemical parameters assessed varied considerably between studies, and no single marker was uniformly applied as a diagnostic standard. Physical therapy methods included passive mobilisation with joint compression, reflex locomotion therapy (RLT), structured motor activity, and combined massage–exercise programmes. Fracture incidence was prespecified as a secondary outcome; however, fractures were inconsistently reported in the included literature and were not systematically categorised by anatomical type such as long-bone or rib fractures, limiting meaningful pooled comparison. Vitamin D Supplementation

Three studies evaluated vitamin D strategies in preterm infants [12–14]. In Alizadeh et al.’s trial of 68 preterm infants (29–38 weeks, 1100–1950 g), daily supplementation with either 400 IU or 1000 IU of vitamin D produced no significant differences in serum calcium, phosphate, or ALP after nine weeks. None showed clinical or radiological signs of rickets, suggesting that 400 IU/day was sufficient to maintain normal bone mineralisation [12].

Kołodziejczyk-Nowotarska et al. compared fixed-dose vitamin D regimens (800–1000 IU/day) with monitored, dose-adjusted regimens in 109 preterm infants (24–33 weeks). Both approaches prevented deficiency; however, serially adjusted dosing, where vitamin D supplementation was repeatedly increased, reduced, or withheld according to scheduled sequential serum 25(OH)D assessments to maintain concentrations within the predefined 20–80 ng/mL safety range, achieved physiological 25(OH)D levels more consistently (93.0% vs. 70.4%; *p* = 0.017) and reduced toxic concentrations (>90 ng/mL) (4.5% vs. 23.8%; *p* = 0.013), although neonatal 25(OH)D interpretation remains constrained by assay-specific variability, residual epimer cross-reactivity, and the absence of universally standardised preterm reference thresholds. The fixed-dose group developed hypervitaminosis D and renal complications, including nephrolithiasis and nephrocalcinosis [13].

### Active vitamin D analogue therapy

Rustico et al. studied a retrospective cohort of 32 infants (<37 weeks) with metabolic bone disease and secondary hyperparathyroidism. Unlike the preceding prophylactic vitamin D studies, this cohort represented a clinically high-risk subgroup with severe established MBD, including prolonged total parenteral nutrition dependence (73%), necrotising enterocolitis or bowel surgery, TPN cholestasis, exposure to bone-adverse medications, and fractures present in 53% of infants. Calcitriol was introduced as adjunctive therapy when standard mineral optimisation was limited or poorly tolerated and PTH exceeded 100 pg/mL. Adjunctive calcitriol therapy (median 0.05 µg/kg/day) significantly reduced PTH and increased calcium, phosphate, and tubular phosphate reabsorption. Transient hypercalcaemia and hypercalciuria resolved with dose adjustment, and no nephrocalcinosis occurred [14].

### Calcium supplementation

Two studies examined calcium supplementation [15, 16]. Shiva et al. compared continuous infusion with intermittent intravenous calcium administration in 78 preterm infants (<32 weeks or <1500 g) receiving parenteral nutrition. Continuous infusion consisted of 4–5 mL/kg/day of 10% calcium gluconate delivered within the 24-h parenteral nutrition solution, whereas intermittent administration consisted of 1–2 mL/kg/dose given three to four times daily as separate boluses. Continuous infusion was associated with lower ALP levels and reduced metabolic bone disease incidence (25% vs. 52.6%; *p* < 0.05), without infusion-related complications [15]. Mean serum calcium, phosphate, vitamin D, and PTH concentrations did not differ significantly; however, elevated ALP levels (>900 IU/L) were less frequent with continuous infusion (25% vs. 52.6%) [15]. However, this strategy requires reliable central venous or PICC access and careful monitoring to avoid calcium-phosphate precipitation, which may limit routine use in some neonatal units. In addition, the bolus group received a lower mean calcium dose, suggesting that improved mineral delivery rather than infusion timing alone may have contributed to the observed benefit.

Similarly, Lee et al. reported that surviving preterm infants with birth weight <1200 g followed over the first 12 weeks of life receiving higher-calcium PN (1.8–2.2 mmol/kg/day) had lower rates of radiological MBD (0% vs. 12.8%; *p* = 0.02) than those receiving 1.3 mmol/kg/day, alongside significantly lower rates of hyperphosphatasaemia defined by peak ALP > 800 IU/L (5% vs. 19.1%) and less prolonged hypophosphataemia, with serum phosphate <1.33 mmol/L persisting for more than 2 weeks in 7.5% versus 42.6% of infants. Serial monitoring further demonstrated consistently lower ALP and higher serum phosphate concentrations across weeks 0–12 in the higher-calcium cohort. Higher calcium and phosphate concentrations were sustained, with no differences in growth outcomes [16].

### Phosphate supplementation

Two studies investigated phosphate supplementation and PN composition [17, 18]. In Angelika et al.’s study, 43% of preterm infants (27–32 weeks) receiving PN without phosphate developed osteopenia, correlating with longer PN duration and raised ALP levels. Each additional PN day increased osteopenia risk (OR 1.12; *p* = 0.018), underscoring importance of early phosphate inclusion [17].

Chang et al. compared PN formulations containing sodium glycerophosphate versus inorganic phosphate in 402 infants. The organic formulation enabled greater calcium–phosphate delivery, resulting in higher serum phosphate, lower ALP, and fewer cases of biochemical osteopenia, defined by reduced biochemical evidence of impaired bone mineralisation including lower peak alkaline phosphatase levels and fewer infants with ALP > 500 U/L, and seizures, though mild hypocalcaemia was more frequent [18]. No significant difference in radiographic MBD incidence was observed.

### Combined calcium–phosphate supplementation

Four studies assessed combined calcium-phosphate strategies [19–22]. Torabi et al. found no significant between-group differences in biochemical or growth outcomes after six weeks of supplementation with calcium gluconate 10% at 5 mL/kg/day (45 mg/kg/day elemental calcium), potassium phosphate 17% at 1 mL/kg/day (24 mg/kg/day elemental phosphorus), and vitamin D (400 IU/day) compared with vitamin D alone, likely due to short duration and suboptimal dosage [19].

Motokura et al. showed that high-phosphate PN in ELBW infants lowered ALP and reduced the need for alfacalcidol (3.8% vs. 64%; *p* < 0.001), without metabolic complications [20]. In Sureshchandra et al.’s large retrospective cohort of 188 extremely preterm infants (<29 weeks’ gestation; Group 1 *n* = 103, Group 2 *n* = 85), a high-mineral parenteral nutrition formulation (Ca:P = 1.3) significantly reduced radiological MBD (47.3% vs. 82.8%; *p* < 0.01) and eliminated severe disease, with higher phosphate and lower ALP associated with improved outcomes [21]. Infants receiving organic phosphate at a Ca:P ratio of 1.3 had lower rates of Grade 1 MBD and no cases of Grades 2 or 3, whereas those given inorganic phosphate at a 1.1 ratio had higher MBD incidence [21].

Körnmann et al. evaluated anthropometric and DXA-derived bone mineralisation outcomes in preterm infants receiving unfortified donor milk, unfortified preterm formula, or early fortified human milk or formula once enteral intake reached ≥50 mL/day [22]. Early enteral fortification increased mineral intake but did not increase bone mineral content (BMC) or bone mineral density (BMD) at term-equivalent age, as measured by whole-body dual-energy X-ray absorptiometry. Phosphorus intake was positively associated with linear growth, calcium intake inversely associated with BMC, and a modest synergistic calcium-phosphate effect on BMD was observed (p = 0.036). Human milk volume was positively correlated with BMC [22]. However, interpretation was limited by the small number of acceptable DXA scans, exclusion of scans with movement artefact, variable gestational age at assessment, and the lack of robust normative DXA reference standards for very preterm infants.

### Physiotherapy

Five RCTs evaluated physiotherapy-based interventions for the prevention and management of MBDP. Interventions included passive mobilisation with joint compression, RLT, structured motor activity, and combined massage–exercise programmes. Across all studies, physiotherapy was well tolerated, with no reported adverse events or detrimental effects on growth.

In a trial involving 29 preterm infants (physiotherapy group *n* = 15, control group *n* = 14) aged 26–34 weeks’ gestation, <1600 g, Vignochi et al. found that daily 15-min motor physiotherapy sessions administered five times a week led to greater daily weight gain (27.4 ± 3.7 vs 21.0 ± 4.4 g/day, *p* < 0.001), length gain (1.28 ± 0.34 vs 0.78 ± 0.23 cm/week, *p* < 0.001), and tibial length gain (0.16 ± 0.04 vs 0.10 ± 0.03 cm/week, *p* = 0.02) compared with standard care. DEXA showed increases in BMC, BMD, and bone area (all p < 0.001). Gains in BMC correlated positively with weight (r = 0.59, *p* = 0.001) and lean mass (r = 0.50, *p* = 0.002), while no significant biochemical differences were observed between groups [23].

Similarly, El-Farrash et al. randomised 36 VLBW infants (≤32 weeks, ≤1500 g) to receive passive range-of-motion exercises or tactile stimulation for four weeks. The exercise group demonstrated greater daily weight gain (34.7 vs 17.6 g/day, *p* < 0.001) and showed a broader improvement in biochemical markers of bone metabolism, including higher serum phosphorus (7.0 vs 4.9 mg/dL, *p* = 0.001), lower alkaline phosphatase (425 vs 543 IU/L, *p* = 0.005), and lower urinary calcium-to-phosphate ratios (88.9 vs 180, *p* = 0.040), while serum calcium, magnesium, and CTX remained comparable between groups. DEXA confirmed higher whole-body bone mineral density (0.5 vs 0.4 g/cm², *p* < 0.001), and severe MBD at discharge occurred less frequently (5.6% vs 38.9%, *p* = 0.009) [24].

Aly et al. evaluated 30 preterm infants (28–35 weeks’ gestation), randomised to receive combined massage and physical activity or standard care. The intervention produced increases in serum calcium from 8.5 ± 1.4 to 10.1 ± 1.2 mg/dL, representing a statistically significant but physiologically modest rise within the neonatal reference range (19.5% vs 4.3%, *p* = 0.002) and the bone formation marker PICP (*p* < 0.001), accompanied by a rise in PTH levels (*p* < 0.001). Serum alkaline phosphatase did not differ significantly between groups over the study period. No between-group differences were found in bone resorption markers (Pyd), suggesting that bone formation was enhanced without increased resorption [25].

Two RCTs by Torró-Ferrero et al. [26, 27] examined the effects of RLT. In the first, 46 preterm infants (29–34 weeks’ gestation) were randomised to RLT, passive mobilisation with joint compression (PMC), or massage for four weeks. RLT resulted in higher tibial speed of sound (SOS) compared with PMC (mean difference 19.6 m/s, *p* = 0.015) and massage (26.7 m/s, *p* = 0.001), without adverse effects on growth [26]. In a subsequent multicentre trial of 106 preterm infants (26–34 weeks’ gestation), RLT and PMC were compared with massage. Time and time–group effects were observed for osteocalcin, with a marked increase in the RLT group (*p* < 0.001) and a smaller rise in the PMC group (p = 0.016). No significant changes occurred in bone resorption markers (NTx or β-CTx). Height gain was greatest in the RLT group (*p* = 0.018), and no intervention-related pain or complications were reported [27].

## Discussion

The evidence highlights that MBDP management requires an integrated approach combining early nutritional optimisation and targeted physiotherapy to improve bone mineralisation and skeletal outcomes in preterm infants.

### Supplementation interventions

Seven studies evaluated calcium and phosphate supplementation. Three demonstrated that higher concentrations of calcium and phosphate formulations were associated with a reduced risk of MBD, one reported no significant association between supplementation dosage and outcomes, and another examined how the method of calcium delivery influenced efficacy. The remaining two studies assessed phosphate-specific or combined feeding effects. Three additional studies investigated isolated vitamin D supplementation. Biochemical markers included ALP, serum phosphate, calcium, PTH, and bone turnover indices, though these varied considerably across studies and did not directly reflect fracture prevention. Fractures were inconsistently reported and not classified by anatomical type.

Sureshchandra et al., Lee et al., Chang et al., and Motokura et al. explored the effects of higher-concentration calcium and phosphate formulations on MBD risk using radiological or biochemical endpoints [16, 18, 20, 21]. These studies highlight that supplementation efficacy depends on formulation composition and the calcium-to-phosphate ratio, aligning with findings by Greer, Lane, and Hall, who demonstrated optimal phosphate retention and calcium absorption when the Ca:P ratio ranges between 1.5:1 and 1.7:1 [28].

Lee et al. found that higher calcium intake was associated with a reduced incidence of MBD [16]. The accompanying biochemical profile supported these findings, suggesting that adequate calcium intake enhances phosphate retention and reduces secondary metabolic disturbances frequently observed in MBD.

Comparable results were reported by Motokura et al., in whom increased calcium and phosphate supplementation was associated with improved biochemical markers and reduced reliance on adjunctive vitamin D analogues, without metabolic complications [20]. Together, these findings support the safety and efficacy of early, balanced mineral supplementation in ELBW infant populations. In contrast, Chang et al. demonstrated no significant difference between biochemical improvement and radiographic MBD, indicating that enhanced mineral delivery does not necessarily translate into structural skeletal benefit [18].

Körnmann et al. highlighted that skeletal development is influenced not only by mineral intake but also by nutrient balance and feeding strategy [22]. The observed relationships between calcium and phosphate suggest that mineral interactions play a critical role in bone accretion. The contribution of human milk volume further emphasises that enteral feeding practices are important determinants of skeletal development in this population.

Although most studies demonstrated benefits of optimised mineral supplementation, results were not entirely consistent. Torabi et al. reported limited benefit from combined calcium and phosphate supplementation, attributed to short intervention duration and suboptimal dosing [19].

The method of mineral delivery also appears clinically relevant. Shiva et al. demonstrated that continuous calcium infusion was associated with more favourable metabolic profiles compared with intermittent bolus administration, suggesting that continuous infusion allows more stable calcium concentrations, more closely replicates intrauterine mineral delivery, and may reduce hormonal fluctuations such as PTH surges that promote bone resorption [15, 29, 30].

Three studies investigated isolated vitamin D supplementation. Findings were consistent with evidence that a daily dose of 400 IU is adequate to prevent OFP in clinically stable preterm infants. Alizadeh et al. found no significant differences in serum ALP, phosphate, or calcium between infants receiving 400 IU and 1000 IU per day, with no clinical or radiological evidence of rickets [12].

Kołodziejczyk-Nowotarska et al. demonstrated that individualised, monitored vitamin D supplementation enhances safety without compromising efficacy, emphasising dosing strategies based on biochemical surveillance rather than fixed regimens. In this randomised trial, preterm infants born at 24 0/7 to 32 6/7 weeks received either fixed-dose vitamin D (800–1000 IU/day) or monitored supplementation adjusted according to serial 25(OH)D levels. Both regimens prevented deficiency but monitored dosing maintained acceptable vitamin D levels more consistently at 52 weeks (93.0% vs. 70.4%; *p* = 0.017) and reduced toxic concentrations >90 ng/mL. Hypercalciuria and nephrolithiasis occurred predominantly in the fixed-dose group, highlighting the risks of unmonitored high-dose supplementation [13].

Rustico et al. evaluated calcitriol therapy in preterm infants with MBD and secondary hyperparathyroidism. Their findings support calcitriol as a targeted adjunctive therapy in selected infants, through suppression of PTH secretion, reduced urinary phosphate wasting, and enhanced mineral absorption. Resolution of metabolic disturbances with dose adjustment indicates that calcitriol can be used safely when carefully monitored [14, 31].

The duration and composition of PN emerged as critical determinants of MBDP risk. Angelika et al. found that prolonged PN without phosphate supplementation markedly increased OFP incidence, with each additional PN day raising OFP risk by 12% and PN beyond 15 days conferring a fivefold higher risk. Elevated ALP levels further supported impaired bone turnover, attributed to early phosphate omission and disruption of the optimal Ca:P ratio [17].

Adverse effects associated with mineral supplementation remain important considerations. Chinoy, Mughal, and Padidela described the potential for nephrocalcinosis, particularly with elevated calcium and suppressed or normal PTH, underscoring the need to maintain balanced Ca:P ratios [6]. Hypercalcaemia, generally defined in preterm neonatal practice as a total serum calcium concentration exceeding approximately 11 mg/dL (2.75 mmol/L), although exact laboratory thresholds may vary, represents another clinically relevant complication, manifesting as irritability, vomiting, constipation, and hypertension, requiring prompt recognition and management to prevent sequelae [32].

### Physiotherapy interventions

Across the five RCTs included in this review, physiotherapy interventions for preterm infants, including passive mobilisation, RLT, structured motor activity, and combined massage–exercise programmes, were associated with improvements in bone mineralisation and biochemical markers of bone metabolism, with more variable effects on somatic growth compared with standard care or tactile stimulation alone. Despite heterogeneity in modality, duration, and intensity, all interventions were delivered to medically stable preterm infants within the neonatal intensive care setting and were reported to be well tolerated without evidence of harm, though tolerability including autonomic stability was not quantified.

Vignochi et al. demonstrated that regular physiotherapy produced greater gains in weight, length, tibial length, BMC, BMD, and bone area compared with controls, with gains in lean mass correlating positively with bone mineral content, suggesting that mineralisation benefits were driven by accrual of lean tissue rather than adiposity [23]. Similarly, El Farrash et al. found that passive range-of-motion exercises reduced the incidence of severe metabolic bone disease at discharge, improved serum phosphate, lowered ALP and urinary calcium-to-phosphate ratios, and enhanced BMD [24]. These skeletal benefits were accompanied by higher daily weight gain but without increases in length or head circumference, indicating preferential improvement in bone mineralisation over linear growth [24]. Aly et al. found that a combined massage and physical activity protocol increased markers of bone formation without changes in resorption markers, suggesting a net anabolic skeletal effect [25].

Two complementary trials by Torro-Ferrero et al. further supported the efficacy of RLT as a potentially superior modality. Their single-centre trial demonstrated that RLT attenuated the decline in tibial SOS over four weeks more effectively than passive mobilisation or massage, reflecting preserved bone quality through sustained mechanical loading [26]. In their subsequent multicentre study, RLT increased osteocalcin levels and height gain relative to massage, while passive mobilisation produced intermediate osteocalcin improvements [27]. Neither RLT nor passive mobilisation altered bone resorption markers or negatively impacted anthropometric growth trends, reinforcing their favourable safety profile [27].

When contextualised within the broader evidence base, these findings align with the Cochrane review by Schulzke et al., which pooled data from 11 RCTs and reported short-term gains in BA, BMC, and daily weight gain at programme completion, with variable and modest effects on linear growth and no significant differences in head circumference [33]. This parallels the results of El Farrash et al. [24], Aly et al. [25], and the Torro-Ferrero trials [26, 27]. Importantly, the Cochrane analysis found that gains were not sustained at 12 months corrected age, implying that benefits are transient unless mechanical stimulation continues beyond discharge [33]. Safety outcomes were concordant, with no reported increases in fractures, retinopathy of prematurity, intraventricular haemorrhage, sepsis, prolonged oxygen dependency, or extended hospital stay [33].

The findings are further supported by Stalnaker and Poskey’s systematic review of 12 RCTs [34], which concluded that early, structured physical activity, particularly passive range-of-motion exercises with gentle joint compression, enhances bone mineralisation and strength when combined with adequate nutrition. Superior outcomes were identified with 15-min sessions, limited additional benefit beyond four weeks, and equivalent efficacy between parent- and therapist-delivered programmes. The lack of additive benefit from combining massage with exercise mirrors Aly et al. [25, 34].

Mehra et al.’s scoping review reinforced these observations, noting that interventions involving mechanical loading consistently improved tibial SOS, BMD, biochemical formation markers, and anthropometric measures such as weight and tibial length [35]. RLT yielded the greatest improvements in bone formation markers relative to passive mobilisation or massage, a finding replicated in the Torro-Ferrero trials [26, 27].

### Limitations

This review has several methodological limitations. The search strategy included selected databases and English-language publications only, with exclusion of studies before 2000, which may have introduced publication bias and led to omission of relevant studies. Some full texts were also inaccessible, limiting data completeness.

Limitations within included studies included poor control of confounding variables and inconsistencies between comparison groups. Angelika et al., Chang et al., El-Farrash et al., and Rustico et al. did not clearly define controlled variables, limiting attribution of findings to the interventions studied [14, 17, 18, 24].

Variation in outcome measures and definitions of MBD, alongside heterogeneity in study design and interventions, limited comparability and hindered meta-analysis. Therefore, results were synthesised qualitatively by intervention type and outcome. Variations in research groups further complicated analysis, as most studies included one control and one intervention group, whereas Körnmann et al. and Torró-Ferrero et al. used one control and two intervention groups [22, 26], increasing analytical complexity and reducing clarity regarding causal relationships.

External and ecological validity were limited. Although Vignochi et al., Aly et al., El-Farrash et al., and Torró-Ferrero et al. reported total sample sizes of *n* = 29, *n* = 30, *n* = 36, and *n* = 46, respectively, the number who actually received the physiotherapy interventions was smaller: Vignochi et al. (*n* = 15), Aly et al. (*n* = 15), El-Farrash et al. (*n* = 18), and Torró-Ferrero et al. (*n* = 31) [23–25, 27]. These reduced intervention-exposed populations further limit representativeness and reduce generalisability. Attrition bias also arose from data loss due to exclusions or deaths; for instance, Chang et al. excluded 26 infants for incomplete data or follow-up loss [18].

Although the interventions used in the studies by Vignochi et al., El-Farrash et al., and Torró-Ferrero et al. were based on the Moyer-Mileur protocol, developed in 1995, its current level of adoption within neonatal intensive care units is not well described [23, 24, 26, 27]. The studies did not specify the training for neonatal therapists delivering the intervention, which limits assessment of intervention fidelity and reduces the generalisability of the findings.

In addition, although no negative overall outcomes were attributed to the interventions, their potential effects on autonomic stability and longer-term neurodevelopment remain uncertain, as these outcomes were not evaluated. Reporting pre- and post-intervention homoeostatic measures would have helped determine their overall impact on infants’ physiological stability. The use of standardised pain and stress scales for neonates prior to, during, and post the therapy intervention would help to further determine the overall safety of this type of intervention.

### Future research

Future research should include large, multicentre RCTs directly comparing combined nutritional and physiotherapy interventions for MBDP. Standardisation of outcome measures, such as biochemical markers, imaging, and follow-up duration, is needed to enable meta-analysis and strengthen recommendations. Standardisation and objective evaluation of therapist training and of the treatment protocol with rigorous reporting of outcomes to include neonatal pain and stress scoring scales and autonomic stability both during and post-treatment would assist with the determination of the overall safety of these interventions with this population. Additional studies should clarify optimal calcium-to-phosphate ratios, dosing strategies for parenteral and enteral nutrition, and safe vitamin D thresholds across gestational ages and clinical conditions. Longitudinal research is also necessary to determine if early physiotherapy yields sustained improvements in bone density and fracture risk into later life.

## Conclusion

This systematic review demonstrated that MBDP is a multifactorial disorder best addressed through early and optimised mineral supplementation. Adequate calcium, phosphate, and vitamin D improved biochemical and radiological outcomes in preterm infants. Although several studies reported improvements in bone mineralisation and growth with interventions such as passive mobilisation or RLT, these physiotherapy approaches were delivered exclusively within controlled research settings, are not standardised or routinely used in neonatal units, and lack evaluation on autonomic stability and longer-term safety and may carry risk. As such, despite potential benefits observed in research contexts, these interventions fall outside the current scope of neonatal therapy services and cannot be recommended for clinical practice.

Early, individualised management remains essential for MBDP prevention. Further research should refine supplementation protocols and rigorously assess the safety, feasibility, and long-term effects of physiotherapy-based interventions before wider implementation is considered.

## Supplementary information

Supplementary Table 1. Characteristics of included studies evaluating supplementation and physiotherapy interventions for metabolic bone disease of prematurity

## Data Availability

All data analysed during this study are included in this published article and its references. No new datasets were generated. Further information is available from the corresponding author upon reasonable request.
