# Supplementary material for: Prevention and management of metabolic bone disease of prematurity: a systematic review
Source: J Perinatol. 2026 Jun 29;46(9):1608–15. doi: 10.1038/s41372-026-02781-z (PMC13588742; doi:10.1038/s41372-026-02781-z)
Supplement: Supplementary file 1 [file 41372_2026_2781_MOESM1_ESM.docx]

**Supplementary Table 1. Characteristics of included studies evaluating supplementation and physiotherapy interventions for metabolic bone disease of prematurity**

| Author, Year | Study design (Oxford level of evidence) | Study Population | Intervention Group | Control Group | Inclusion and Exclusion Criteria | Outcomes (primary and secondary) | Key Results | Intervention Type |
| --- | --- | --- | --- | --- | --- | --- | --- | --- |
| Rustico et al., 2014 | 3b (Case Control) | 32 preterm infants (<37 weeks’ gestation) with metabolic bone disease (MBD) and secondary hyperparathyroidism (PTH >100 pg/mL) | Calcitriol treatment (enteral or intravenous); median dose 0.08 µg/kg/day for median 207 days | None | Inclusion Criteria:  • GA < 37 weeks  • Radiographic evidence of bone demineralisation  • PTH > 100 pg/mL  • Calcitriol treatment between 1–12 months after birth  Exclusion Criteria:  • Significant renal insufficiency  • Suspected vitamin D metabolism or parathyroid hormone defect  • No post-treatment PTH monitoring | Association of calcitriol therapy with biochemical markers of bone disease (PTH, calcium, phosphate, alkaline phosphatase, TRP, urine Ca/Cr) | • Significant decrease in PTH (220 → 25 pg/mL, p < 0.001)  • Increase in serum calcium (9.9 → 10.3 mg/dL, p < 0.001)  • Increase in phosphate (4.3 → 5.4 mg/dL, p = 0.001)  • Increase in TRP (81 → 91.5%, p = 0.03)  • No significant change in alkaline phosphatase  • Transient hypercalcemia with hypercalciuria in 3/32, resolved after dose reduction | Supplementation |
| Shiva et al., 2024 | 1b (RCT) | 78 preterm infants (<32 weeks or <1500g) | Continuous infusion of calcium gluconate (4-5ml/kg/day) (n=40) | Intermittent bolus administration (1-2ml/kg/dose 3-4 times/day) (n=38) | Inclusion Criteria:  • gestational age at birth <32 weeks or birth weight < 1500g.  Exclusion Criteria:  • infants with severe asphyxia, chromosomal abnormalities, congenital metabolic abnormalities, malignancy  • maternal history of hyperparathyroidism and/or vitamin D disorders | Primary: Incidence of MBDP based on serum ALP > 900 IU/L and serum phosphorus < 5.6 mg/dL.  Secondary: Serum Ca, phosphate, ALP, vitamin D, and PTH levels. | Day 45 mean serum ALP significantly higher in intermittent bolus group (988.2 vs 753.2 IU/L, p=0.031).  Significantly more infants in intermittent bolus group met criteria for MBDP diagnosis (20/38 vs 10/40, p<0.05). | Supplementation |
| Körnmann, 2017 | 1b (RCT) | 109 preterm infants with BW <1500g | Group C (n = 37): Mother’s own milk (MOM) + preterm formula + early fortification (>50 mL/day from day 1) | Group A (n = 40): MOM + donor milk (unfortified) Group B (n = 32): MOM + preterm formula (unfortified) | Inclusion Criteria:  • preterm infants with a weight <1500g  • parental written consent before first enteral feed  Exclusion Criteria:  • congenital malformations  • congenital infection proven within 72hr after birth  • perinatal asphyxia with a pH <7.0  • maternal drugs and/or alcohol use during pregnancy  • any intake of cow's milk based products prior to randomisation | Growth (weight, length) and bone mineralization at term corrected age (TCA) measured by dual-energy X-ray absorptiometry (DXA) | • No significant differences in weight, length, BMC, or BMD between groups.  • Phosphorus intake positively associated with length (p=0.048).  • Calcium intake negatively associated with BMC (p<0.001).  • Human milk volume positively associated with BMC (p=0.017).  • Interaction between Ca and P only for BMD (p=0.036).  • Higher early Ca and P intake did not improve bone mineralization at TCA. | Supplementation |
| Lee, 2022 | 2b (Retrospective Cohort) | 87 premature infants with BW <1200g | High calcium group (n=40): Parenteral nutrition containing ~2 mmol/kg/day calcium (1.8–2.2 mmol/kg/day) and 1.7 mmol/kg/day phosphorus using sodium glycerophosphate (organic phosphate) | Low calcium group (n=47): Parenteral nutrition containing 1.3 mmol/kg/day calcium and 1.3 mmol/kg/day phosphorus using inorganic phosphate | Inclusion Criteria:  • premature infants with birth weight less than 1200g  Exclusion Criteria:  • any baby that died before end of study  • infants transferred to another hospital | • Incidence of radiological metabolic bone disease (MBD)  • Incidence of hyperphosphatasia (ALP >800 IU/L)  • Incidence and duration of hypophosphataemia (serum phosphate <1.33 mmol/L)  • Longitudinal ALP, calcium, phosphate levels (0–12 weeks)  • Growth (length z-score from birth to 37 weeks) | • Radiological MBD: 0/40 (High Ca) vs 6/47 (Low Ca), p=0.02  • Fractures: 0 vs 1 (ns)  • ALP >800 IU/L: 2/40 vs 9/47, p=0.048  • Hypophosphataemia >2 weeks: 3/40 vs 20/47, p=0.0002  • ALP significantly lower and serum calcium/phosphate higher across 12 weeks (ALP significantly lower (p < 0.0001))  • No difference in drop in length z-score (p=0.288)  • No adverse events or nephrocalcinosis reported | Supplementation |
| Motokura, 2021 | 2b (Retrospective Cohort) | • 48 extremely low-birth-weight (ELBW) infants (<1000 g)  • 22 infants in low-phosphorus (P) group  • 26 infants in high-phosphorus (P) group | High-P group (n = 26):  • Received parenteral calcium (Ca) and phosphorus (P) simultaneously from day 1 via double-lumen catheter.  • Mean daily PN intake (first week): Ca = 32.7 mg/kg/day; P = 17.3 mg/kg/day.  • Amino acids = 2.1 g/kg/day. | Low-P group (n = 22):  • Received Ca initially (40–64 mg/kg/day) with delayed P (days 2–4), not given concurrently.  • Mean daily PN intake (first week): Ca = 21.7 mg/kg/day; P = 9.0 mg/kg/day.  • Amino acids = 1.8 g/kg/day. | Inclusion criteria:  • ELBW infants (<1000 g) born and admitted to Kyoto University Hospital between 2011–2017.  Exclusion criteria:  • In-hospital death  • Transfer to another facility  • Cholestasis (direct bilirubin > 2 mg/dL)  • Major congenital or chromosomal abnormalities  • Congenital bone or neuromuscular disease | Primary outcomes:  • Biochemical markers of MBD – serum alkaline phosphatase (ALP), serum Ca, serum P, urinary Ca:Cr ratio (uCa/Cr), and tubular reabsorption of phosphate (%TRP).  • Incidence of secondary hyperparathyroidism (ALP > 1300 IU/L, P < 5 mg/dL, %TRP ≤ 93%).  Secondary outcomes:  • Radiological changes (Koo grade ≥ 2).  • Requirement for alfacalcidol therapy.  • Incidence of bone fractures. | • Mean daily Ca and P intake significantly higher in high-P group (Ca p = 0.001; P p < 0.001).  • Serum ALP significantly higher in low-P group (1685 vs 1270 IU/L; p = 0.014).  • Alfacalcidol use: 64% in low-P vs 3.8% in high-P (p < 0.001).  • No difference in serum Ca, P, or %TRP.  • Trend toward fewer x-ray changes (23% → 7.7%; p = 0.141).  • No bone fractures reported.  • Conclusion: High-P intake enabled higher Ca intake, reduced ALP, and decreased need for alfacalcidol without adverse skeletal effects. | Supplementation |
| Sureshchandra, 2025 | 2b (Retrospective Cohort) | 188 preterm infants <29w GA | PN2 group (n=85) – received parenteral nutrition with higher calcium and phosphate concentrations (Ca 2.3 mmol/kg/day, P 1.8 mmol/kg/day; Ca:P ratio 1.3:1 | PN1 group (n=103) – received standard PN formulation with lower calcium and phosphate (Ca 1.6 mmol/kg/day, P 1.4 mmol/kg/day; Ca:P ratio 1.1; inorganic phosphate) | Inclusion:  • Infants born <29 weeks’ gestation and admitted to NICU within 24 h of birth.  Exclusion:  • Infants admitted >24 h after birth.  • Discharged/transferred/died before 6-week MBDP screening.  • Born during washout period (Jan–May 2018). | •Primary Outcome: Incidence and severity of MBDP (radiologically graded 0–3).  •Secondary Outcomes: Serum Ca, phosphate, ALP, PTH, vitamin D, TRP at 6 weeks; diagnostic sensitivity/specificity of biomarkers; adverse events (hypercalcemia, hypophosphatemia, nephrocalcinosis). | • MBDP incidence decreased from 82.8% (77/93) in PN1 to 47.3% (27/57) in PN2.  • Grade 2–3 MBDP eliminated in PN2 group (14% → 0%, p<0.01).  • Serum phosphate <1.5 mmol/L: sensitivity 79%, specificity 77%.  • ALP >500 U/L: sensitivity 72%, specificity 71%.  • Higher Ca and P levels during PN in PN2 group (p<0.01).  • No increase in nephrocalcinosis (23% vs 28%, p=0.99).  • No hypocalcemia in PN2; mild hypercalcemia increase without adverse outcomes. | Supplementation |
| Torabi, 2014 | 1b (RCT) | 40 premature neonates (<37 weeks, <2500g) | Calcium + Phosphorus + Vitamin D supplements (n=20) | Vitamin D 400 units daily only (n=20) | Inclusion Criteria:  • GA <37 wks, BW <2500g  • medically stable  • only breastfed  Exclusion Criteria:  • neonates w/ TPN and NPO for >1wk, receiving medications interfering w/ Vitamin D metabolism  • infants sepsis syndrome, signifciant respiratory, neurologic, renal, genetic, cardiovascular, hepatic or GI diseases  • maternal osteomalacia, diabetes, parathyroid gland disease and renal disorders | Radiological evidence of osteopenia and biochemical markers. | No significant radiological changes of osteopenia in treatment group (40% vs 65%, p<0.113).  No significant effects on calcium, phosphorus, and alkaline phosphatase levels. | Supplementation |
| Angelika, 2021 | 2b (Prospective cohort study) | 30 preterm infants (<32 weeks, <1500g) | All infants received PN containing calcium but no phosphate supplementation | No comparison group (observational only) | Inclusion Criteria:  • GA <32 weeks  • BW <1500g  Exclusion Criteria:  • Multiple congenital anomalies  • Congenital heart defects with fluid restriction  • Asphyxia  • Maternal diabetes  • Long-term corticosteroid (>2 weeks), furosemide (>2 weeks), or aminophylline (>1 month) use  • Death before bone mineralization measurement  • Withdrawal or no parental consent | Primary Outcome:  • Incidence of osteopenia of prematurity (OFP) based on wrist radiographs (Koo’s grading).  • Serum ALP levels at start, end of PN, and discharge.  Secondary Outcome:  • Relationship between PN duration and OFP incidence. | • Incidence of OFP: 43% (13/30 infants).  • Duration of PN: Longer in OFP group (16 vs 12 days, p=0.018).  • PN >15 days ↑ risk of OFP (OR 5.40; 95% CI 1.12–26.04; p=0.035).  • ALP levels: Increased over time and were higher in OFP group (429±260 vs 252±72 U/L; p=0.01).  • Positive correlation between PN duration and ALP levels (ρ=0.488; p=0.006). | Supplementation |
| Chang, 2025 | 2b (Retrospective Cohort) | 402 preterm neonates (<37 weeks) receiving PN for ≥7 days | Sodium glycerophosphate (SGP) used in parenteral nutrition (n = 205) | Inorganic phosphate (IP) used in parenteral nutrition (n = 197) | Inclusion Criteria:  • gestational age of less than 37 weeks  • admitted to the neonatal intensive care unit of Changhua Christian Children’s Hospital  • received parenteral nutrition for at least 7 days  • time period: between January 2019 and September 2021  Exclusion Criteria:  52 patients were excluded  • 26 for whom data were incomplete or who were lost to follow-up after discharge  • 14 had gastrointestinal tract malformations requiring surgery, complex congenital heart disease, or chromosomal abnormalities  • 12 died before discharge | Primary outcomes:  • Metabolic bone disease (MBD) of prematurity.  • Serum calcium, phosphate, ALP, creatinine.  • Growth parameters (weight, length, head circumference) up to 1 year.  Secondary outcomes:  • Incidence of seizures, AKI, BPD, electrolyte imbalance (hypocalcemia, hypophosphatemia, etc.), ROP, NEC, IVH. | • Calcium & phosphate content in PN significantly higher with SGP across all 4 weeks.  • Serum phosphate ↑ (4.0 ± 1.2 vs. 3.5 ± 1.3 mg/dL, p = 0.001).  • Serum ALP ↓ (402.8 ± 202.8 vs. 466.4 ± 228.6 U/L, p = 0.004).  • % ALP >500 ↓ (24.6% vs. 37.6%, p = 0.007).  • Hypocalcemia ↑ (41.5% vs. 31.5%, p = 0.038).  • Seizure incidence ↓ (4.9% vs. 13.2%, p = 0.003).  • No difference in MBD, growth, or other morbidities (ROP, NEC, IVH, BPD, AKI). | Supplementation |
| Alizade, 2006 | 1b (RCT) | 68 preterm infants (<38 weeks, <2000g) | Group A: 400 IU/day vitamin D (n=32) | Group B: 1000 IU/day vitamin D (n=36) | Inclusion Criteria:  • Gestational age <38 weeks  • Birth weight <2000 g  Exclusion Criteria:  • Maternal use of anticonvulsants, diuretics, corticosteroids  • Maternal diabetes mellitus  • Previous IUGR/SGA baby  • Infant on furosemide chronically or NPO >2 weeks  • Failure to comply with supplementation/radiography | • Primary outcomes: Serum calcium, phosphate, and alkaline phosphatase at 9 weeks; clinical and radiological evidence of rickets  • Secondary outcomes: Anthropometric measurements, duration of supplementation | • No significant differences in mean serum calcium (9.83 vs 9.71 mg/dL, p=0.326), phosphate (5.55 vs 5.44 mg/dL, p=0.466), or alkaline phosphatase (602.5 vs 633.3 IU/L, p=0.147).  • No radiological or clinical evidence of rickets in either group.  • Four infants (two in each group) had wide fontanelle without other rickets signs; one had hypothyroidism.  • Concluded that 400 IU/day vitamin D was sufficient for prevention of osteopenia of prematurity. | Supplementation |
| Kołodziejczyk-Nowotarska, 2021 | 1b (RCT) | 109 preterm infants (24-32 weeks) | Monitored vitamin D therapy with dose adjustment (n=54) | Standard vitamin D therapy (500 IU/day + nutrition, ~800-1000 IU/day) (n=55) | Inclusion Criteria:  • preterm infants born between 24 0/7–32 6/7 weeks gestational age  • admitted within 48 hours postnatally  • caregivers provided written informed consent  Exclusion Criteria:  • gestational age >32 6/7 weeks  • infants with major congenital abnormalities, cholestasis, or severe illness incompatible with survival at birth  • lack of consent or communication difficulties with caregivers | Primary:  • Number of neonates with deficient or excess 25(OH)D at 40 ± 2 weeks PCA.  Secondary:  • 25(OH)D at 35 and 52 weeks PCA  • Incidence of MBD (osteopenia)  • Vitamin D toxicity, hypercalcemia / hypercalciuria  • Nephrolithiasis and SOS (Z-score) | • At 52 weeks PCA, a higher percentage of monitored infants had safe vitamin D levels (93% vs 70%; p = 0.017).  • Mean 25(OH)D lower in monitored vs control (50.6 ± 18.3 vs 62.2 ± 25.3 ng/mL; p = 0.03).  • 23% of control group had to stop supplementation due to toxic levels (≥90 ng/mL).  • Vitamin D toxicity biochemical markers in 2 control infants and nephrolithiasis in 5 infants with very high levels.  • No significant difference in MBD incidence (4–12%), bone mass (SOS Z-score), or TRP indices between groups.  • Monitored group avoided excess vitamin D without increasing deficiency risk. | Supplementation |
| Aly et al, 2004 | 1b (Double-blind randomized controlled trial) | 30 preterm infants (28-35 weeks) | • Combined massage + passive physical activity  • Daily sessions until infants reached 1.8 kg  • 5 repetitions of flexion–extension and compression at wrists, elbows, shoulders, knees, hips, and ankles  • Gentle stroking massage (around 1 min per body region, prone position)  • Vital signs monitored before, during, and after | Standard care (n=15) | Inclusion:  • GA ≤ 35 weeks  • Postnatal age < 2 weeks  • Tolerating full enteral feeds  • Not receiving medications except vitamin supplementation  Exclusion:  • Congenital malformations  • Asphyxia  • Musculoskeletal, hepatic, or renal disease | Biochemical markers of bone metabolism: serum PICP (bone formation), urinary Pyd (bone resorption), serum calcium, alkaline phosphatase, and PTH. | • increase PICP in intervention (62.5 to 73.8 ng/mL, p < 0.01) vs decrease PICP in control (82.3 to 68.8 ng/mL, p < 0.01); between-group p < 0.001 to increase bone formation  • increase Serum Ca greater in intervention ( +19.5 % vs +4.3 %, p = 0.002 )  • increase PTH in intervention ( 8.3 → 15.1 pg/mL ) vs ↓ in control ( 33 → 25.7 pg/mL ), p < 0.001  • Pyd increased in both groups, no between-group difference , no increase in resorption  • ALP unchanged in both groups | Physiotherapy |
| El-Farrash et al., 2019 | 1b (RCT) | 36 VLBW preterm infants (≤32 weeks, ≤1500g) | • Passive range-of-motion exercises with gentle compression, flexion, and extension of all limb joints (shoulder, elbow, wrist, hip, knee, ankle) (n=18)  • Duration: 10 min/day, 5 days/week for 4 weeks  • Based on Moyer-Mileur protocol | • Tactile stimulation only (holding and stroking without joint movement) (n=18)  • Same duration and schedule as intervention  • Standard neonatal care and nutrition provided | Inclusion Criteria:  • Preterm infants ≤ 32 weeks’ gestation  • Birth weight ≤ 1500 g and appropriate for GA  • Postnatal age ≤ 1 week  • Medically stable and not receiving physiotherapy for other indications  Exclusion Criteria:  • Major congenital or acquired anomalies (respiratory, cardiac, neural-tube, abdominal-wall, chromosomal)  • Grade III–IV IVH or septic shock  • Major surgery within first 2 weeks of life  • Use of diuretics, methylxanthines, or corticosteroids | • Anthropometric measurements (weight, length, OFC, rate of gain)  • Biochemical markers of bone mineralisation (Ca, PO₄, Mg, ALP, urinary Ca/PO₄ ratio, serum CTX)  • Radiological assessments via DEXA (BMC, BMD, bone area, lean mass, fat mass)  Secondary outcomes:  • Feeding tolerance and time to full enteral feeds  • Length of hospital stay  • Complications of prematurity  • Adverse effects of exercise protocol | • Increase in median daily weight gain in the exercise group (34.7 vs 17.6 g/day, p < 0.001)  • Increase in whole-body BMD (0.5 vs 0.4 g/cm², p < 0.001)  • Increase in serum phosphate (p = 0.001)  • Decrease in ALP (p = 0.005)  • Decrease in urinary Ca/PO₄ ratio (p = 0.04)  • Decrease in neonatal morbidity (p < 0.006)  • No significant change in serum Ca, Mg, or CTX  • No adverse events reported | Physiotherapy |
| Torró-Ferrero et al., 2022a | 1b (RCT) | 46 preterm infants (29-34 weeks) | RLT (reflex locomotion therapy) group (n=17) - 16 min/day (2 × 8 min sessions), 5 days/week for 4 weeks.  PMC (passive mobilization with joint compression) group (n=14) | Control group received massage (n=15) | Inclusion Criteria:  • Preterm infants in NICU  • Clinically stable and on full enteral feeds  • Born between 29 and 34 weeks GA  Exclusion Criteria:  • Grade III–IV IVH or NEC requiring surgery  • Congenital malformations or metabolic/genetic disease  • Mechanical ventilation during full enteral feeding  • Use of diuretics/corticosteroids or presence of bone fractures | Primary Outcome: Tibial Speed of Sound (Tibial-SOS; marker of bone mineralisation) measured by quantitative ultrasound.  Secondary Outcomes: Weight, length, and head circumference (Z-scores). | • Tibial-SOS significantly higher in RLT group vs control (mean difference 26.66, p = 0.001) and vs PMC group (mean difference 19.6, p = 0.015).  • No significant differences between PMC and massage groups.  • All groups showed some loss of bone mineralisation, least pronounced in RLT group.  • No adverse effects or fractures reported. | Physiotherapy |
| Torró-Ferrero et al., 2022b | 1b (RCT) | 106 preterm infants (26–34 weeks’ GA), medically stable and on full enteral feeds | • RLT group (n = 38) – Reflex locomotion therapy 16 min/day (2 × 8 min sessions), 5 days/week for 4 weeks  • PMC group (n = 32) – Passive mobilisation with gentle joint compression 15 min/day × 5 days/week × 4 weeks | Massage control group (n = 36) – 15 min/day gentle massage, 5 days/week for 4 weeks | Inclusion criteria:  • Gestational age 26–34 weeks  • Haemodynamically stable  • Receiving full enteral nutrition  Exclusion criteria:  • Neurological, metabolic or genetic disorders  • Severe IVH (Grades III–IV)  • BPD, mechanical ventilation, or NEC requiring surgery  • Fractures or use of diuretics/corticosteroids | • Primary: Bone biomarkers – osteocalcin (OC), NTx (serum & urine), and β-CTx  • Secondary: Anthropometric measures (weight, length, head circumference) | • Significant increase in osteocalcin (OC) in the RLT group vs PMC and control (p = 0.013).  – increased OC (61.6 to 99.7 ng/mL, p < 0.001); EGpmc increased (p = 0.016); CG no change.  • No significant differences in NTx (urine/serum) or β-CTx between groups.  • Height significantly increased in RLT group (p = 0.018); weight and head circumference increased similarly in all groups.  • No adverse events or pain observed (NIPS ≤ 2). | Physiotherapy |
| Vignochi et al., 2008 | 1b (RCT) | 29 preterm infants (GA 26–34 weeks, BW <1600 g) admitted to NICU | Motor physical therapy for 15 min daily, 5×/week until discharge (n = 15). Passive range-of-motion and gentle joint compressions on upper/lower limbs and thorax, based on the Moyer-Mileur protocol. | Standard care (n = 14): routine nursing and maternal care without structured passive movement or compressions. | Inclusion:  • Preterm infants (GA 26–34 wks, BW <1600 g)  • Clinically stable and appropriate for GA  • No associated disease other than prematurity  • Tolerating full enteral feeds (around 110 kcal/kg/day)  Exclusion:  • Severe periventricular haemorrhage (Grade III–IV)  • Severe sepsis  • Use of diuretics or corticosteroids  • Need for mechanical ventilation | Bone mineralization (BMC, BMD, bone area) and growth parameters (weight, length, lean/fat mass). | The PT group had significantly greater:  • Daily weight gain: 27.4 ± 2.4 g vs 21.0 ± 4.4 g (p < 0.001)  • Length gain: 1.3 ± 0.3 cm/week vs 0.8 ± 0.2 cm/week (p < 0.001)  • BMC gain: 434 ± 247.5 mg vs –8.9 ± 11.4 mg (p < 0.001)  • BMD gain: 8.4 ± 5.6 mg/cm² vs –3.1 ± 5.5 mg/cm² (p < 0.001)  • Bone area gain: 10.3 ± 5.0 cm² vs 1.5 ± 2.0 cm² (p < 0.001)  • Lean mass gain: 271 ± 21 g vs 109 ± 12 g (p < 0.009).  No significant difference in fat mass (p = 0.43). Serum Ca, P, and PTH unchanged. | Physiotherapy |

**Abbreviations:**

MBD = metabolic bone disease
 MBDP = metabolic bone disease of prematurity
 PN = parenteral nutrition
 ALP = alkaline phosphatase
 PTH = parathyroid hormone
 TRP = tubular reabsorption of phosphate
 BMD = bone mineral density
 BMC = bone mineral content
 RCT = randomized controlled trial
 ELBW = extremely low birth weight
 VLBW = very low birth weight
 DXA = dual-energy X-ray absorptiometry
 GA = gestational age
 BW = birth weight

Levels of evidence classified according to the Oxford Centre for Evidence-Based Medicine criteria.
